# Choose your own TALE: Two novel cell death-inducers identified by engineered effectors

**DOI:** 10.1093/plphys/kiae228

**Published:** 2024-04-25

**Authors:** Manuel González-Fuente

**Affiliations:** Assistant Features Editor, Plant Physiology, American Society of Plant Biologists; Faculty of Biology & Biotechnology, Ruhr-University Bochum, D-44801 Bochum, Germany

Genome editing has broken into the mainstream: a Nobel prize, huge potential in medicine and agriculture, ethical debates, obsolete regulations, famous controversies.… From a mechanistic point of view, genome editing techniques are just clever ways to utilize natural cellular processes to modify genomic sequences in a targeted manner. For instance, the famous CRISPR-Cas9 is based on the ability of certain bacteria to recognize and degrade viral DNA. Before CRISPR-Cas9 became “a thing,” scientists had developed another genome editing technique using bacterial transcription activator-like effector proteins (TALEs) from *Xanthomonas*. These bacteria translocate TALEs directly into the plant nucleus, where they bind specific sequences of the plant DNA and induce the expression of downstream genes (Perez-Quintero and Szurek 2019). This recognition of the DNA is achieved by the modular structure of TALEs, whose central domain contains tandem repeats, each one binding a single nucleotide. The recognition specificity comes from the amino acid composition of 2 key residues in the tandem repeats, which determine the nucleotide each repeat will bind to (Boch et al. 2009). Different *Xanthomonas* species have evolved different TALEs to activate the expression of a wide variety of susceptibility genes to the bacterial benefit (Zhang et al. 2022). In turn, plants have also evolved mechanisms to coopt the TALEs transcriptional activation to induce the expression of so-called executor genes that cause a protective form of cell death that prevents the pathogen proliferation (Nowack et al. 2022).

In this issue of *Plant Physiology*, Roeschlin et al. (2024) exploited the modularity of TALEs to ingeniously identify 2 novel executor genes in *Nicotiana benthamiana*. Taking advantage of one of the shortest TALEs able to cause cell death (Roeschlin et al. 2019), the authors engineered random artificial TALEs with increased length in their DNA-binding domain to narrow the search in the genome of the executor genes responsible for the cell death (Fig.). Together with transcriptomic data, they revealed a patatin-like protein and a nectarin-like protein as the targets of the TALE that induce cell death in *N. benthamiana*. Interestingly, proteins from neither of these 2 families were previously characterized as TALE targets nor as regulators of immunity/cell death, reinforcing previous notions that cell death can be achieved by overexpression of genes otherwise involved in normal cellular processes (Nowack et al. 2022).

**Figure.**
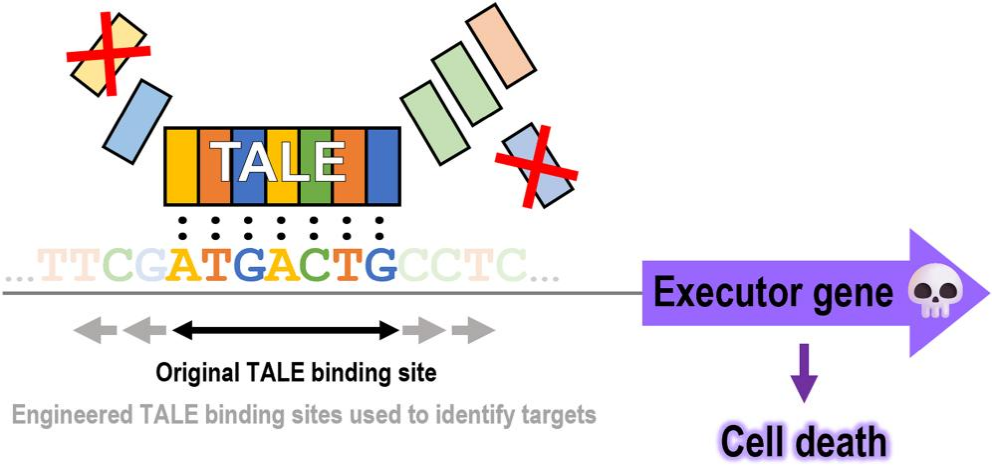
Engineered TALEs with extended tandem repeats eases the identification of target executor genes. The identification of TALE targets is complicated because in silico prediction tools yield many false positives, especially for short TALEs with short binding sites. To ease the identification of bona fide targets of a short cell death-inducing TALE, random addition of tandem repeats was fused to the N or C terminus. Evaluating the maintenance or loss of the ability to cause cell death of these artificial extended TALEs, together with transcriptomic data, allows narrowing the list of putative candidates and identifying the causative executor genes.

Since the DNA-binding specificity code of TALEs was deciphered more than a decade ago (Boch et al. 2009), one could assume that the in silico prediction of the target(s) of a given TALE would be easy. But as biologists, we are all aware that theory and practice rarely go hand in hand. So, even if there are currently several algorithms designed to predict TALE targeting, they often yield many false positives and miss already known validated targets (Erkes et al. 2019). Thus, extensive laboratory work is still required for the identification and functional validation of bona fide targets of TALEs. The work by Roeschlin et al. (2024) proves that engineered TALEs can hasten this process, discovering 2 novel *N. benthamiana* executors genes as a proof-of-concept. Interestingly, these executor genes are structurally and functionally distinct from any other previously known resistance gene. These findings not only prove that a single TALE can activate the expression of 2 different and seemingly additive executor genes, but they also broaden the catalogue of genes with roles in plant immunity, opening the doors for their possible exploitation in resistance breeding programs in the future.
